# A “Good” Smoke? The Off-Label Use of Cannabidiol to Reduce Cannabis Use

**DOI:** 10.3389/fpsyt.2022.829944

**Published:** 2022-03-17

**Authors:** Davide Fortin, Vincent Di Beo, Sophie Massin, Yann Bisiou, Patrizia Carrieri, Tangui Barré

**Affiliations:** ^1^University Paris 1 Sorbonne, Paris, France; ^2^Aix Marseille University, INSERM, IRD, SESSTIM, Sciences Economiques & Sociales de la Santé & Traitement de l'Information Médicale, ISSPAM, Marseille, France; ^3^Artois University, CNRS, IESEG School of Management, University of Lille, UMR 9221, Lille Economie Management, Arras, France; ^4^University Paul Valéry Montpellier 3, CORHIS, Montpellier, France

**Keywords:** cannabidiol (CBD), cannabis (marijuana), cannabis use disorder (CUD), smoking, France, harm reduction

## Abstract

**Background:**

Although cannabis use is common in France, it is still criminalized. Cannabidiol (CBD) products, including CBD-rich cannabis, are legally available. Although previous results suggested that CBD may have benefits for people with cannabis use disorder, there is a lack of data on cannabis users who use CBD to reduce their cannabis consumption. We aimed to identify (i) correlates of this motive, and (ii) factors associated with successful attempts to reduce cannabis use.

**Methods:**

A cross-sectional online survey among French-speaking CBD and cannabis users was conducted. Logistic regressions were performed to identify correlates of using CBD to reduce cannabis consumption and correlates of reporting a large reduction.

**Results:**

Eleven percent (*n* = 105) of our study sample reported they primarily used CBD to reduce cannabis consumption. Associated factors included smoking tobacco cigarettes (adjusted odds ratio (aOR) [95% confidence interval (CI)] 2.17 [1.3–3.62], *p* = 0.003) and drinking alcohol (aOR [95%CI] 1.8 [1.02–3.18], *p* = 0.042). Of these 105, 83% used CBD-rich cannabis to smoke, and 58.7% reported a large reduction in cannabis consumption. This large reduction was associated with non-daily cannabis use (aOR [95%CI] 7.14 [2.4–20.0], *p* < 0.001) and daily CBD use (aOR [95%CI] 5.87 [2.09–16.47], *p* = 0.001). A reduction in cannabis withdrawal symptoms thanks to CBD use was the most-cited effect at play in self-observed cannabis reduction.

**Conclusions:**

Cannabis use reduction is a reported motive for CBD use—especially CBD-rich cannabis to smoke—in France. More studies are needed to explore practices associated with this motive and to accurately assess CBD effectiveness.

## Introduction

Cannabis use is being increasingly liberalized worldwide (1), and cannabidiol (CBD) products are proliferating (2). Recent trends in Europe and the U.S. suggest an increase in the prevalence of cannabis use disorders (CUD) (3–5), for which there is still no approved pharmaceutical treatment. Preliminary data have highlighted that CBD has benefits in CUD treatment (6). Evidence is also growing that nabiximols—an oromucosal spray providing a balanced mixture of tetrahydrocannabinol (THC) and CBD—brings benefits in CUD treatment (7–11). However, little is known about cannabis users who use CBD to reduce their cannabis use (12).

Cannabis use is still criminalized in France, including for therapeutic purposes. Users may be punished by up to 1 year in prison and a fine of 3,750 € (13); since 2020, an on-the-spot fine of 150 € can replace the normal procedure at the police's discretion (14). Despite this criminalization, France has the highest prevalence of cannabis use among young people and adults in Europe (15), and indicators of cannabis use disorder and treatment for dependence are on the rise (16). The demand for herbal cannabis is also growing, as is its potency (17). A similar trend in increasing potency has been observed internationally (18, 19).

Despite strong development of the CBD market internationally—including in France—in recent years (2), the legal status of CBD products still remains unclear. Recent rulings by the Court of Justice of the European Union (20) and the French Court of Cassation (21) confirmed that CBD products legally produced in the European Union can be sold in France. The legal status of cannabis flowers with <0.2% of THC—which are widely marketed in France—is still unclear.

Given this context, we aimed to investigate whether some French CBD users consume this phytocannabinoid to reduce cannabis consumption, and to identify potential correlates for this motive. We also aimed to document the pattern of CBD use associated with this motive, and to describe the effects at play in reducing cannabis consumption, as reported by users.

## Materials and Methods

### Data Collection

An online survey written in French was conducted using a Google form between April 23, 2020 and March 30, 2021. The protocol followed the guidelines of the Declaration of Helsinki, and the INSERM Ethics Committee provided ethical approval (approval #20-677 dated April 23, 2020). A link to the survey was distributed *via* media outlets specializing in cannabis-based products, CBD user groups on Facebook, and a community of people with chronic health conditions. Inclusion criteria for the present study were: previous-month CBD use and lifetime illegal cannabis use.

In the survey, the acronym “CBD” was used to include all legal products marketed as containing a significant amount of CBD, irrespective of their actual CBD content. This therefore covered legal CBD-rich THC-low (<0.2%) cannabis to smoke (called “CBD-rich cannabis” in this manuscript), as opposed to “regular” high THC cannabis (called “illegal cannabis” here). The survey collected self-reported data on the following: socio-demographic and substance use (cannabis, CBD, alcohol, tobacco) characteristics, preferred mode of CBD use, and primary reason for CBD use. The latter was collected using the question “In the past 30 days, why have you used CBD?”. Only one answer was allowed from a list of options which included “to reduce the use of tobacco or other substances (illegal cannabis, alcohol, etc.)” (Supplementary Table 1). People who ticked this answer were then asked if they used CBD for illegal cannabis use reduction. Those who replied “yes”, were then asked (i) to what extent CBD had an impact on their illegal cannabis use (“large reduction/moderate reduction/no effect/moderate increase/large increase/I do not know”; these answer options were dichotomized into “large reduction” vs. “no large reduction” (i.e., all other answers)), and (ii) which CBD-related effects were involved in reducing their illegal cannabis use (“In your opinion, what CBD-related effects were at play in reducing your illegal cannabis use?”). Participants could choose several responses from the following four pre-determined options: “using less illegal cannabis in a joint,” “longer time between smoking two joints of illegal cannabis.” “reduction in illegal cannabis withdrawal symptoms,” and “longer time before smoking first joint of the day”.

### Outcomes

Two principal outcomes were built. The first was “using CBD for illegal cannabis use reduction”, as regarded the whole of the study sample. The second was “reporting a large reduction in cannabis consumption thanks to CBD use”, and regarded only the sub-sample of respondents who answered “yes” to the question for the first outcome.

### Statistical Analyses

**Figure 1:**
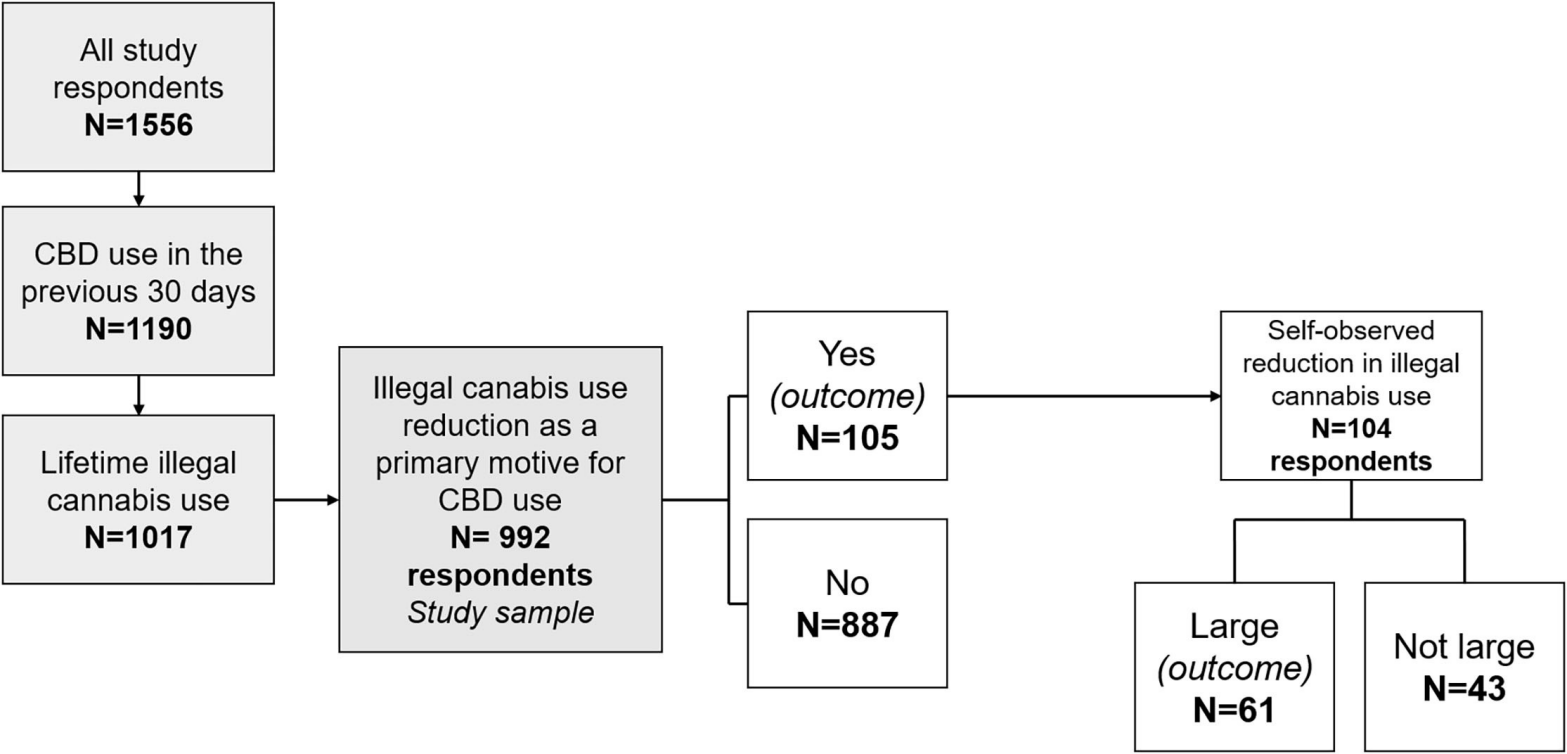
Flow chart of the study, design of the regression outcomes, and distribution of participants according to the two study outcomes. CBD, cannabidiol.

We characterized users using CBD as a means to reduce their illegal cannabis use by comparing their socio-demographic and socio-behavioral characteristics with the rest of the sample using a Chi-square (categorical variables) or Wilcoxon's (continuous variables) test. We then performed a logistic regression with “having used CBD to reduce illegal cannabis use” as an outcome and socio-demographic and behavioral characteristics as explanatory variables (Figure 1). For the sub-population who reported this reason, we performed a second logistic regression with “reporting a large reduction in illegal cannabis following CBD use” as the outcome, and variables related to CBD use as explanatory variables (Figure 1). For both regressions, only variables with a liberal *p*-value < 0.20 in the univariable analyses were considered eligible for the multivariable model. The final multivariable model was built using a backward stepwise procedure. The likelihood ratio test (*p* < 0.05) was used to define the variables to maintain in the final model.

We also provided a description of the self-reported CBD-related effects at play to reduce illegal cannabis consumption, and used Chi-square tests to compare these effects between the group of participants reporting a large reduction in illegal cannabis use and those who did not.

## Results

Among the 1,556 respondents, 1,190 participants used CBD in the 30 days before the survey (Figure 1). Of the 1,017 of the latter who reported lifetime illegal cannabis use, 992 answered the question related to the primary reason why they used CBD. Study sample characteristics are provided in Table 1. Our study sample consisted in 992 CBD (and lifetime illegal cannabis) users. Most were men (74.5%), median age was 34 years, and most resided in France (96.5%). Over 10% (10.6% (*n* = 105)) reported having used CBD in the previous month primarily to reduce their illegal cannabis consumption. The vast majority of the study sample (99.4%) had used illegal cannabis before their first use of CBD. Among those who reported illegal cannabis reduction as their primary reason to use CBD, 66.7 and 8.8% had also used it for tobacco and alcohol use reduction, respectively.

**Table 1:** Study sample socio-demographic and behavioral characteristics and factors associated with the use of cannabidiol to reduce cannabis consumption (logistic regression).

	**CBD use to reduce or stop cannabis use**			**Univariable analyses**		**Multivariable analysis (*****n*** **= 964)**	
	**No** ***n* = 887** **(89.4%)**	**Yes** ***n* = 105 (10.6%)**	***p*** **-value^a^**	**OR (95% CI)**	***p*** **-value**	**aOR (95% CI)**	***p*** **-value**
	***N*** **(%)**	***N*** **(%)**					
**Gender (*****n*** **= 984)**			0.673				
Male	653 (74.3)	80 (76.2)		1			
Female	226 (25.7)	25 (23.8)		0.9 (0.56–1.45)	0.673		
**Age (years)** median (IQR)	35 (28–42)	30 (25–37)	<0.001	0.95 (0.93–0.97)	<0.001	0.95 (0.93–0.97)	<0.001
**Smoking tobacco cigarettes in previous 30 days** **(*****n*** **= 982)**			0.012				
No	306 (34.8)	21 (20.6)		1		1	
Yes	504 (57.3)	80 (78.4)		2.31 (1.4–3.82)	0.001	2.17 (1.3–3.62)	0.003
Smoking mainly e-cigarettes	70 (8.0)	1 (1.0)		0.21 (0.03–1.57)	0.128	0.23 (0.03–1.79)	0.162
**Alcohol consumption in previous 30 days (*****n*** **= 973)**			0.012				
No	247 (28.4)	17 (16.7)		1		1	
Yes	624 (71.6)	85 (83.3)		1.98 (1.15–3.4)	0.013	1.8 (1.02–3.18)	0.042
**Cannabis use prior to first CBD use**			0.398				
No	6 (0.7)	0 (0)		-			
Yes	881 (99.3)	105 (100)		-			
**High educational level**^b^ **(*****n*** **= 954)**			0.484				
No	281 (32.9)	36 (36.4)		1			
Yes	574 (67.1)	63 (63.6)		0.86 (0.56–1.32)	0.485		
**Having a job** **(*****n*** **= 990)**			0.063				
No	640 (72.2)	84 (80.8)		1		1	
Yes	246 (27.8)	20 (19.2)		0.62 (0.37–1.03)	0.065	0.51 (0.29–0.89)	0.017
**Housing**			0.917				
Owner	301 (33.9)	33 (31.4)		1			
Tenant	433 (48.8)	55 (52.4)		1.16 (0.73–1.83)	0.527		
Living with parents or friends	111 (12.5)	12 (11.4)		0.99 (0.49–1.98)	0.968		
Prefer not to respond	42 (4.7)	5 (4.8)		1.09 (0.4–2.94)	0.871		
**Self-reported income level** ^c^			0.394				
Below average	301 (33.9)	30 (28.6)		1			
Average	412 (46.4)	56 (53.3)		1.36 (0.85–2.18)	0.194		
Above average	174 (19.6)	19 (18.1)		1.1 (0.6–2)	0.767		
**Body mass index** **(*****n*** **= 984)**			0.022				
<25 kg/m^2^	634 (72.0)	85 (82.5)		1			
≥25 kg/m^2^ (overweight or obesity)	247 (28.0)	18 (17.5)		1.84 (1.08–3.12)	0.024		
**Daily cannabis use in previous 30 days** **(*****n*** **= 978)**			0.213				
No	701 (80.2)	78 (75.0)		1			
Yes	173 (19.8)	26 (25.0)		1.36 (0.83–2.24)	0.227		
**Used CBD to reduce tobacco use in previous 30 days** **(*****n*** **= 105)**^d^			-				
No^e^	-	35 (33.3)		-			
Yes	-	70 (66.7)		-			
**Used CBD to reduce alcohol use in previous 30 days** **(*****n*** **= 102)**^d^			-				
No^e^	-	93 (91.2)		-			
Yes	-	9 (8.8)		-			

a *Chi-square (categorical variables) or Wilcoxon rank-sum test (continuous variables)*.

b *Higher educational level was defined as attending third-level education*.

c *Income level was subjectively assessed using participant-perceived average income level as a reference value*.

d *This question was answered only by users who declared that reducing substance use was their primary reason to use CBD. This variable was not included in the regression model and is displayed for descriptive purposes only*.

e *Percentages are given for users who declared reducing cannabis use as the primary reason to use CBD*.

*aOR, adjusted odds ratio; CBD, cannabidiol; CI, confidence interval*.

In multivariable analysis, declaring to use CBD primarily to reduce illegal cannabis consumption was associated with younger age, tobacco cigarette smoking in the previous month, alcohol drinking in the previous month, and not having a job (Table 1).

Among those who declared using CBD to reduce their illegal cannabis, half (51.5%) had used CBD for less than a year, and 38.5% had used it every day in the previous month (Table 2). Sixty-one (58.7%) reported that their CBD use led to a large reduction in illegal cannabis consumption, 36 (34.6%) a moderate reduction, 6 (5.8%) no reduction, and 1 (1.0%) a moderate increase (1 missing value). Most (84.3%) smoked CBD-rich cannabis, while only 7.8% administered it orally (Table 2). A large majority (94.0%) of those who smoked CBD-rich cannabis mixed it into joints (i.e., together with tobacco or illegal cannabis).

**Table 2:** Cannabidiol products pattern of use among users who used cannabidiol to reduce illegal cannabis consumption, and factors associated with a large reduction in illegal cannabis consumption.

	**Large reduction in illegal cannabis use**			**Univariable analyses**		**Multivariable analysis (*****n*** **= 103)**	
	**No** ***n* = 43 (41.4%)**	**Yes** ***n* = 61 (58.6%)**	***p*** **-value^a^**	**OR (95% CI)**	***P*** **-value**	**aOR (95% CI)**	***P*** **-value**
	***N*** **(col %)**	***N*** **(col %)**					
**Time since first CBD use**			0.858				
Less than a year	21 (48.8)	32 (53.3)		1			
Between 1 and 2 years	10 (23.3)	14 (23.3)		0.92 (0.34–2.45)	0.865		
More than 2 years	12 (27.9)	14 (23.3)		0.77 (0.3–1.97)	0.581		
**Daily CBD use in previous 30 days**			<0.001				
No	36 (83.7)	28 (45.9)		1		1	
Yes	7 (16.3)	33 (54.1)		6.06 (2.34–15.73)	<0.001	5.87 (2.09–16.47)	0.001
**CBD purchase locations in previous 30 days**			0.326				
On the internet	12 (27.9)	12 (19.7)		1			
Other	31 (72.1)	49 (80.3)		1.58 (0.63–3.96)	0.328		
**Principal mode of CBD administration in previous 30 days**			0.213				
Smoked (combustion)	37 (88.1)	49 (81.7)		1			
Inhalation	1 (2.4)	7 (11.7)		5.29 (0.62–44.85)	0.127		
Other (infusion, ingestion)	4 (9.5)	4 (6.7)		0.76 (0.18–3.22)	0.704		
**CBD price per gram during most recent purchase**			0.569				
<5€	10 (25.6)	12 (20.7)		1			
Between 5 and 9€	20 (51.3)	36 (62.1)		1.5 (0.55–4.08)	0.427		
10€ or more	9 (23.1)	10 (17.2)		0.93 (0.27–3.17)	0.902		
**Previous month CBD budget**			0.287				
<40€	9 (21.4)	14 (24.1)		1			
Between 41 and 100€	23 (54.8)	23 (39.7)		0.64 (0.23–1.78)	0.395		
More than 100€	10 (23.8)	21 (36.2)		1.35 (0.44–4.16)	0.601		
**Daily cannabis use in the previous 30 days**			<0.001				
No	23 (53.5)	54 (90)		1		1	
Yes	20 (46.5)	6 (10.0)		0.13 (0.05–0.36)	<0.001	0.14 (0.05–0.42)	<0.001
**Smoked pure CBD** ^b^			0.046				
No^c^	36 (100)	43 (89.6)		-			
Yes	0 (0)	5 (10.4)		-			

a *Chi-square (categorical variables) or Wilcoxon rank-sum test (continuous variables)*.

b *This question was answered only by users who declared smoking CBD. This variable was not included in the regression model and is displayed for descriptive purposes only*.

c *Percentage is given for users who declared smoking as their principal mode of CBD administration in previous 30 days*.

*aOR, adjusted odds ratio; CBD, cannabidiol; CI, confidence interval*.

In multivariable analysis, declaring a large reduction (vs. no large reduction) was associated with daily CBD use in the previous month, and non-daily use of illegal cannabis (Table 2).

The self-reported CBD-related effect involved in illegal cannabis use reduction most frequency cited was “reducing cannabis withdrawal symptoms” (44.2%), followed by “delaying first illegal cannabis joint of the day” (24.0%), “using less illegal cannabis in joints” (21.2%) and “increasing the time between smoking joints” (16.3%) (Table 3). Participants reporting a large reduction in illegal cannabis use were more likely to quote “reducing cannabis withdrawal symptoms” as an effect (*p* <10^−3^) but less likely to report “delaying first illegal cannabis joint of the day” (*p* = 0.008; Table 3).

**Table 3:** Self-reported CBD-related effects at play in cannabis use reduction.

	**All**	**Large cannabis use reduction**		
	***n*** **= 104**	**No** ***n* = 43 (41.4%)**	**Yes** ***n* = 61 (58.7%)**	***p*** **-value^a^**
**Using less illegal cannabis in a joint**				0.057
No	82 (78.8)	30 (69.8)	52 (85.2)	
Yes	22 (21.2)	13 (30.2)	9 (14.8)	
**Longer delay between two joints of illegal cannabis**				0.601
Non	87 (83.7)	35 (81.4)	52 (85.2)	
Yes	17 (16.3)	8 (18.6)	9 (14.8)	
**Reduction in illegal cannabis withdrawal symptoms**				<0.001
No	58 (55.8)	34 (79.1)	24 (39.3)	
Yes	46 (44.2)	9 (20.9)	37 (60.7)	
**Longer time before smoking first joint of the day**				0.008
No	79 (76.0)	27 (62.8)	52 (85.2)	
Yes	25 (24.0)	16 (37.2)	9 (14.8)	

a *Chi-square test*.

## Discussion

In a sample of 992 CBD and lifetime illegal cannabis users mostly based in France, we found that using CBD to reduce illegal cannabis use was associated with tobacco smoking, alcohol use and not having a job. Moreover, a large self-reported reduction in illegal cannabis reduction was associated with daily CBD use and non-daily use of illegal cannabis. Finally, in users who used CBD to reduce illegal cannabis consumption, the most common route of CBD administration was smoking (84.3% of all respondents). A reduction in cannabis withdrawal symptoms was the most quoted self-reported CBD-related effect involved in cannabis use reduction (44.2%).

We found that among French CBD and illegal cannabis users, polysubstance use (tobacco and alcohol) is associated with the motivation to reduce illegal cannabis consumption. Interestingly, most of those who reported this motive also reported using CBD to try to cut down or stop tobacco use (few had done so for alcohol use). This would suggest that these CBD users commonly try to reduce their overall smoking (i.e., cannabis and tobacco) behavior. This is very interesting, given that both products are commonly co-consumed (22), and that the continued use of one substance is a barrier to reducing or quitting the other (23) (something already documented for polysubstance use (24, 25)).

The positive association between not having a job and desire to cut down on/stop cannabis consumption through CBD use may seem counter-intuitive given that cannabis is frequently used to cope with stress, and that unemployment is linked with stress. Two hypotheses can be made to explain this association. The first is that the desire to reduce cannabis use may be the result of losing one's job because of cannabis use (26, 27). The second is that unemployed persons may desire to cut down on cannabis-related expenditures because of financial difficulties. Indeed, previous work highlighted that unemployed cannabis buyers were more likely to spend a larger part of their income on cannabis (28). However, as a large majority of the whole study sample was unemployed, this result we found may also be a consequence of biased participant sampling.

We found that a large reduction in illegal cannabis consumption was associated with daily CBD use, which suggests a dose-dependent effect of CBD. This relationship was not observed for high CBD doses (400 and 800 mg) in a phase 2a placebo-controlled randomized trial (6). However, it is possible that having multiple intakes per day enables users to maintain stable CBD plasma levels—and physiological effects—throughout the day. After inhalation, CBD plasma peak is attained within 10 min, with a half-life of ~30 h (29). Moreover, the fact that non-daily illegal cannabis users were more likely to declare a large reduction in cannabis use suggests that the higher the frequency of cannabis use, the more difficult it is to change one's cannabis use pattern; this is probably related to cannabis dependence. In studies elsewhere, the frequency of cannabis flower use was associated with problematic cannabis use (30), the frequency of high-potency cannabis use predicted greater dependence (31), and greater monthly THC exposure was associated with more symptoms of dependence (32).

A few elements in our analysis suggest that CBD-rich cannabis was partially substituted for illegal cannabis in our study sample. First, in the group that used CBD to reduce illegal cannabis use, a majority smoked CBD-rich cannabis. Second, only 6% of the latter smoked “pure” (i.e., non-mixed) CBD-rich cannabis, which means that in almost all cases, it was mixed with either tobacco or illegal cannabis. Third, over 20% of the sub-sample which used CBD to reduce their illegal use declared that it helped them use less illegal cannabis in their joints.

The substitution practice mentioned above should be considered in the context of cannabis use disorder treatment and associated psychiatric outcomes. For example, replacing high-potency cannabis with CBD-rich cannabis would mean a reduction in THC exposure while preserving the gesture and the sensory dimensions of cannabis use. This reduction would likely reduce anxiety and depression in people with cannabis use disorder (33, 34), and be an acceptable and therefore achievable treatment goal for treatment-seeking users (35). Such a reduction in exposure to THC may also lead to cannabis abstinence. These various possibilities need to be clinically tested.

The dominance of smoked CBD-rich cannabis (i.e., as opposed to oral intake) in our study sample is of particular interest, as in France, CBD-rich cannabis is the only cannabis legally available. The global tendency of rising THC levels (i.e., higher potency) and decreasing CBD levels in illegal cannabis (17–19) comes fuel concerns over cannabis use-related harms, as THC is the compound responsible for cannabis use disorder (36, 37). Highly-potent cannabis consumption has been associated with higher risks of cannabis use problems and anxiety disorders (38) as well as psychosis (39, 40). Conversely, CBD seems to attenuate THC-related psychotic-like effects, memory problems (especially in light users), paranoia, anxiety and cannabis-related psychological wellbeing impairment (41–44). This could be due to functional interactions between THC and CBD (45). However, more research is needed to fully elucidate how CBD influences the effects of THC (46). Low-potency cannabis has been described as being one way to reduce cannabis-related health risks (avoiding daily use and combusted cannabis inhalation being two other ways) (47). Accordingly, for cannabis users in France—a population which must choose between illegal high-potency and legal CBD-rich cannabis—mixing both products may be a way for them to create low-risk cannabis, or to move toward creating a “smoking version” of nabiximols. Accordingly, Gibson et al., in the U.S., found that THC + CBD chemovar (9% THC, 10% CBD, from local and legal dispensary) was associated with similar levels of positive subjective effects, but significantly less paranoia and anxiety, as compared to the THC-dominant chemovar (44).

A recent U.S. study also found that CBD and cannabis co-users reported a high proportion of CBD smoking administration (48).

Given that CBD-rich cannabis is sold as the same type of product (i.e., in herbal form) as illegal cannabis, it can be incorporated into one's smoking habits. Moreover, results from previous studies on tobacco smokers suggested that the sensations which smoking creates in the airways contribute to short-term satisfaction, the rewarding effect, and reduced craving (49–51). One can therefore suppose that smoking CBD-rich cannabis may be “beneficial” as part of a strategy to lower exposure to THC: by preserving the smoking-related airway sensation as well as the terpene-related taste (52–54), a minimal reduction in the satisfaction experienced from the act of smoking may be derived from THC-low cannabis as compared to THC-high cannabis (44). In reality, smoking cannabis exposes persons to harmful substances, including carcinogens (55–57). This route of administration is therefore inadvisable, in favor of smoke-free inhalation (58) or oromucosal administration (29).

Our study has several limitations. First, the non-representativeness of our sample of cannabis users in France limits the generalizability of our results, and highlights the need for study duplication. For instance, participants with no job appeared over-represented. Second, we had no data to enable us to detect cannabis use disorder in our sample. However, we did have frequency of use data, which is a good proxy for problematic and low-risk cannabis use (47, 59, 60). Third, we used self-assessed changes (reduction/no change/increase) in cannabis use and had no data on the contextual elements of these changes. Accordingly, we were not able to deduce to what extent CBD was clinically useful in attempts to cut down on cannabis use. Finally, data on the levels of CBD in products consumed by the participants were not available, which limits the solidity of our conclusions. CBD content is highly variable among different products, including cannabis flowers (61–63). For instance, in a large Italian study on THC-low cannabis products, authors found a mean CBD concentration of 4% in the sub-sample (*n* = 185) of flowers with a THC level under 0.2% (i.e., which would be legal in France), with a strong linear correlation between CBD and THC concentrations (personal communication from (64)). As in the survey “CBD” refers to all CBD-based products irrespective of their actual CBD content, answers given by participants may refer to the use of CBD-low products (e.g., THC-low CBD-low legal cannabis flowers or oil with low CBD concentration). Therefore, the effects we reported should be cautiously attributed to CBD-rich cannabis/products.

The main strength of our study is the explorative and original nature of the data; while the use of CBD and CBD-rich cannabis has previously been reported for opioid and pain medication substitution in people with fibromyalgia (65), and the use of nabiximols clinically investigated elsewhere (7), to the best of our knowledge, the substitution of illegal cannabis with CBD has not been previously investigated.

Our findings have many implications. First, we found that some CBD users in France are using the phytocannabinoid in an “off-label” fashion to reduce their illegal cannabis consumption. Further studies should be implemented to confirm and quantify to what extent CBD or nabiximols can in fact accomplish this task. Second, in countries where cannabis use is criminalized but not CBD-rich cannabis, the latter may represent an acceptable tool for THC-related harm reduction. With this in mind, any ban on smokable CBD products could reduce the number of consumers able to reduce their illegal cannabis consumption through CBD use. Bans could also prevent people who smoke cannabis for therapeutic purposes from adjusting their THC/CBD ratio to optimize benefits (62). Finally, non-smoking (e.g., oromucosal) routes of CBD administration to users who wish to reduce their cannabis consumption should be promoted to reduce health-related risks.

To conclude, CBD is used by some illegal cannabis users in France—especially alcohol and tobacco co-users—who wish to reduce their cannabis consumption. In our study, CBD was mainly smoked (i.e., CBD-rich cannabis), and seemed to contribute to cannabis use reduction by lowering cannabis withdrawal symptoms. More studies are needed to explore practices associated with CBD use to reduce cannabis consumption, and to accurately assess its effectiveness.

## Data Availability Statement

The raw data supporting the conclusions of this article will be made available by the authors, without undue reservation.

## Ethics Statement

The study was reviewed and approved by INSERM Ethics Committee provided ethical approval (approval #20-677 dated April 23, 2020). Written informed consent for participation was not required for this study in accordance with the national legislation and the institutional requirements.

## Author Contributions

DF designed the study, interpreted the data, and reviewed the manuscript. VD performed the statistical analyses. SM, YB, and PC designed the study and reviewed the manuscript. TB interpreted the data and wrote the manuscript draft and reviewed it. All authors contributed to the article and approved the submitted version.

## Conflict of Interest

The authors declare that the research was conducted in the absence of any commercial or financial relationships that could be construed as a potential conflict of interest.

## Publisher's Note

All claims expressed in this article are solely those of the authors and do not necessarily represent those of their affiliated organizations, or those of the publisher, the editors and the reviewers. Any product that may be evaluated in this article, or claim that may be made by its manufacturer, is not guaranteed or endorsed by the publisher.

## References

[1] Melchior M Nakamura A Bolze C Hausfater F Khoury FE Mary-Krause M Silva MAD. Does liberalisation of cannabis policy influence levels of use in adolescents and young adults? A systematic review and meta-analysis. BMJ Open. (2019) 9:880. 10.1136/bmjopen-2018-025880 31296507 PMC6624043

[2] Walker LA Koturbash I Kingston R ElSohly MA Yates CR Gurley BJ. Cannabidiol (CBD) in dietary supplements: perspectives on science, safety, and potential regulatory approaches. J Diet Suppl. (2020) 17:493–502. 10.1080/19390211.2020.1777244 32543246

[3] United Nations Office on Drugs Crime. World Drug Report 2020, Second Booklet: Drug Use and Health Consequences. Vienna: UNODC (2020). Available online at: https://wdr.unodc.org/wdr2020/field/WDR20_Booklet_2.pdf (accessed September 14, 2021).

[4] Manthey J. Cannabis use in Europe: current trends and public health concerns. Int J Drug Policy. (2019) 68:93–6. 10.1016/j.drugpo.2019.03.006 31030057

[5] Hasin DS Shmulewitz D Sarvet AL. Time trends in US cannabis use and cannabis use disorders overall and by sociodemographic subgroups: a narrative review and new findings. Am J Drug Alcohol Abuse. (2019) 45:623–43. 10.1080/00952990.2019.1569668 30870044 PMC6745010

[6] Freeman TP Hindocha C Baio G Shaban NDC Thomas EM Astbury D. Cannabidiol for the treatment of cannabis use disorder: a phase 2a, double-blind, placebo-controlled, randomised, adaptive Bayesian trial. Lancet Psychiatry. (2020) 7:865–74. 10.1016/S2215-0366(20)30290-X 32735782 PMC7116091

[7] Lintzeris N Mills L Dunlop A Copeland J Mcgregor I Bruno R. Cannabis use in patients 3 months after ceasing nabiximols for the treatment of cannabis dependence: results from a placebo-controlled randomised trial. Drug Alcohol Depend. (2020) 215:108220. 10.1016/j.drugalcdep.2020.108220 32768992

[8] Trigo JM Soliman A Quilty LC Fischer B Rehm J Selby P. Nabiximols combined with motivational enhancement/cognitive behavioral therapy for the treatment of cannabis dependence: a pilot randomized clinical trial. PLoS ONE. (2018) 13:e0190768. 10.1371/journal.pone.0190768 29385147 PMC5791962

[9] Allsop DJ Copeland J Lintzeris N Dunlop AJ Montebello M Sadler C. Nabiximols as an agonist replacement therapy during cannabis withdrawal: a randomized clinical trial. JAMA Psychiatry. (2014) 71:281–91. 10.1001/jamapsychiatry.2013.3947 24430917

[10] Allsop D Lintzeris N Copeland J Dunlop A McGregor I. Cannabinoid replacement therapy (CRT): nabiximols (Sativex) as a novel treatment for cannabis withdrawal. Clin Pharmacol Therap. (2015) 97:571–4. 10.1002/cpt.109 25777582

[11] Lintzeris N Bhardwaj A Mills L Dunlop A Copeland J McGregor I. Nabiximols for the treatment of cannabis dependence: a randomized clinical trial. JAMA Intern Med. (2019) 179:1242–53. 10.1001/jamainternmed.2019.1993 31305874 PMC6632121

[12] Zobel F Notari L Schneider E Rudmann O. Cannabidiol (CBD): Analyse de Situation. [Rapport de recherche]. Lausanne: Addiction Suisse (2019). Available online at: https://idpc.net/fr/publications/2019/02/cannabidiol-cbd-analyse-de-situation (accessed February 13, 2022).

[13] Massin S Carrieri MP Roux P, De. jure decriminalisation of cannabis use matters: some recent trends from France. Int J Drug Policy. (2013) 24:634–5. 10.1016/j.drugpo.2013.04.008 23726900

[14] LOI n° 2019-222 du 23 mars 2019 de programmation 2018-2022 et de réforme pour la justice. (2019). Available online at: https://www.legifrance.gouv.fr/dossierlegislatif/JORFDOLE000036830320/ (accessed July 02, 2021).

[15] European Monitoring Centre for Drugs Drug Addiction. European Drug Report 2019: Trends and Developments. Lisbon: EMCDDA (2019). Available online at: https://www.emcdda.europa.eu/publications/edr/trends-developments/2019_en (accessed July 12, 2021).

[16] Observatoire Français des Drogues et des Toxicomanies. Drugs, Key Data 2019. OFDT. (2019). Available online at: https://en.ofdt.fr/publications/drugs-key-data/8th-edition-june-2019/ (accessed July 12, 2021).

[17] Gandilhon M Spilka S Masson C. Les mutations du marché du cannabis en France (transformations of French cannabis market). OFDT (2019). Available online at: https://www.ofdt.fr/publications/collections/rapports/thema/les-mutations-du-marche-du-cannabis-en-france-thema/ (accessed April 27, 2021).

[18] Chandra S Radwan MM Majumdar CG Church JC Freeman TP ElSohly MA. New trends in cannabis potency in USA and Europe during the last decade (2008-2017). Eur Arch Psychiatry Clin Neurosci. (2019) 269:5–15. 10.1007/s00406-019-00983-5 30671616

[19] Freeman TP Craft S Wilson J Stylianou S ElSohly M Forti MD. Changes in delta-9-tetrahydrocannabinol (THC) and cannabidiol (CBD) concentrations in cannabis over time: systematic review and meta-analysis. Addiction. (2021) 116:1000–10. 10.1111/add.15253 33160291

[20] Court of Justice of the European Union. In Case C-663/18, REQUEST for a Preliminary Ruling Under Article 267 TFEU from the Cour d'appel d'Aix-en-Provence (Court of Appeal, Aix-en-Provence, France), made by Decision of 23 October 2018, received at the Court on 23 October 2018, in the Criminal Proceedings Against BS, CA. Available online at: https://curia.europa.eu/juris/document/document.jsf?text=&docid=233925&pageIndex=0&doclang=EN&mode=lst&dir=&occ=first&part=1&cid=15340648 (accessed April 27, 2021).

[21] Cour de cassation, criminelle Chambre, criminelle, 23 juin, 2021, 20-84.212. Publié au bulletin (2021). Available online at: https://www.legifrance.gouv.fr/juri/id/JURITEXT000043711045?isSuggest=true (accessed July 1, 2021).

[22] Agrawal A Budney AJ Lynskey MT. The co-occurring use and misuse of cannabis and tobacco: a review. Addiction. (2012) 107:1221–33. 10.1111/j.1360-0443.2012.03837.x 22300456 PMC3397803

[23] Voci S Zawertailo L Baliunas D Masood Z Selby P. Is cannabis use associated with tobacco cessation outcome? An observational cohort study in primary care. Drug Alcohol Depend. (2020) 206:107756. 10.1016/j.drugalcdep.2019.107756 31786396

[24] McCabe SE West BT. The three-year course of multiple substance use disorders in the united states: a national longitudinal study. J Clin Psychiatry. (2017) 78:e537–44. 10.4088/JCP.16m10657 28406266 PMC5453813

[25] Crummy EA O'Neal TJ Baskin BM Ferguson SM. One is not enough: understanding and modeling polysubstance use. Front Neurosci. (2020) 14:e00569. 10.3389/fnins.2020.00569 32612502 PMC7309369

[26] Airagnes G Lemogne C Meneton P Plessz M Goldberg M Hoertel N. Alcohol, tobacco and cannabis use are associated with job loss at follow-up: findings from the CONSTANCES cohort. PLoS ONE. (2019) 14:e0222361. 10.1371/journal.pone.0222361 31498849 PMC6733456

[27] Okechukwu CA Molino J Soh Y. Associations between marijuana use and involuntary job loss in US-representative longitudinal and cross-sectional samples. J Occup Environ Med. (2019) 61:21–8. 10.1097/JOM.0000000000001463 30256305 PMC6314892

[28] Wilkins C Sweetsur P. Individual dollar expenditure and earnings from cannabis in the New Zealand population. Int J Drug Policy. (2007) 18:187–93. 10.1016/j.drugpo.2006.11.002 17689365

[29] Lucas CJ Galettis P Schneider J. The pharmacokinetics and the pharmacodynamics of cannabinoids. Br J Clin Pharmacol. (2018) 84:2477–82. 10.1111/bcp.13710 30001569 PMC6177698

[30] Steeger CM Hitchcock LN Bryan AD Hutchison KE Hill KG Bidwell LC. Associations between self-reported cannabis use frequency, potency, and cannabis/health metrics. Int J Drug Policy. (2021) 97:103278. 10.1016/j.drugpo.2021.103278 34062287 PMC8585676

[31] Freeman TP Winstock AR. Examining the profile of high-potency cannabis and its association with severity of cannabis dependence. Psychol Med. (2015) 45:3181–9. 10.1017/S0033291715001178 26213314 PMC4611354

[32] van der Pol P Liebregts N Brunt T van Amsterdam J de Graaf R Korf DJ. Cross-sectional and prospective relation of cannabis potency, dosing and smoking behaviour with cannabis dependence: an ecological study. Addiction. (2014) 109:1101–9. 10.1111/add.12508 24628797

[33] Mooney LJ Zhu Y Yoo C Valdez J Moino K Liao J-Y. Reduction in cannabis use and functional status in physical health, mental health, and cognition. J Neuroimmune Pharmacol. (2018) 13:479–87. 10.1007/s11481-018-9813-6 30284156 PMC6293461

[34] Hser Y-I Mooney LJ Huang D Zhu Y Tomko RL McClure E. Reductions in cannabis use are associated with improvements in anxiety, depression, and sleep quality, but not quality of life. J Subst Abuse Treat. (2017) 81:53–8. 10.1016/j.jsat.2017.07.012 28847455 PMC5607644

[35] Lozano BE Stephens RS Roffman RA. Abstinence and moderate use goals in the treatment of marijuana dependence. Addiction. (2006) 101:1589–97. 10.1111/j.1360-0443.2006.01609.x 17034438

[36] Kroon E Kuhns L Hoch E Cousijn J. Heavy cannabis use, dependence and the brain: a clinical perspective. Addiction. (2020) 115:559–72. 10.1111/add.14776 31408248 PMC7027478

[37] Zehra A Burns J Liu CK Manza P Wiers CE Volkow ND. Cannabis addiction and the brain: a review. J Neuroimmune Pharmacol. (2018) 13:438–52. 10.1007/s11481-018-9782-9 29556883 PMC6223748

[38] Hines LA Freeman TP Gage SH Zammit S Hickman M Cannon M. Association of high-potency cannabis use with mental health and substance use in adolescence. JAMA Psychiatry. (2020) 77:1044–51. 10.1001/jamapsychiatry.2020.1035 32459328 PMC7254445

[39] Forti MD Quattrone D Freeman TP Tripoli G Gayer-Anderson C Quigley H. The contribution of cannabis use to variation in the incidence of psychotic disorder across Europe (EU-GEI): a multicentre case-control study. Lancet Psychiatry. (2019) 6:427–36. 10.1016/S2215-0366(19)30048-3 30902669 PMC7646282

[40] Di Forti M Marconi A Carra E Fraietta S Trotta A Bonomo M. Proportion of patients in south London with first-episode psychosis attributable to use of high potency cannabis: a case-control study. Lancet Psychiatry. (2015) 2:233–8. 10.1016/S2215-0366(14)00117-5 26359901

[41] Morgan CJA Gardener C Schafer G Swan S Demarchi C Freeman TP. Sub-chronic impact of cannabinoids in street cannabis on cognition, psychotic-like symptoms and psychological well-being. Psychol Med. (2012) 42:391–400. 10.1017/S0033291711001322 21798112

[42] Morgan CJA Schafer G Freeman TP Curran HV. Impact of cannabidiol on the acute memory and psychotomimetic effects of smoked cannabis: naturalistic study: naturalistic study [corrected]. Br J Psychiatry. (2010) 197:285–90. 10.1192/bjp.bp.110.077503 20884951

[43] Morgan CJA Freeman TP Hindocha C Schafer G Gardner C Curran HV. Individual and combined effects of acute delta-9-tetrahydrocannabinol and cannabidiol on psychotomimetic symptoms and memory function. Transl Psychiatry. (2018) 8:181. 10.1038/s41398-018-0191-x 30185793 PMC6125482

[44] Gibson LP Karoly HC Ellingson JM Klawitter J Sempio C Squeri JE. Effects of cannabidiol in cannabis flower: implications for harm reduction. Addict Biol. (2022) 27:e13092. 10.1111/adb.13092 34467598 PMC9357513

[45] Boggs DL Nguyen JD Morgenson D Taffe MA Ranganathan M. Clinical and preclinical evidence for functional interactions of cannabidiol and Δ9-Tetrahydrocannabinol. Neuropsychopharmacology. (2018) 43:142–54. 10.1038/npp.2017.209 28875990 PMC5719112

[46] Freeman AM Petrilli K Lees R Hindocha C Mokrysz C Curran HV. How does cannabidiol (CBD) influence the acute effects of delta-9-tetrahydrocannabinol (THC) in humans? A systematic review. Neurosci Biobehav Rev. (2019) 107:696–712. 10.1016/j.neubiorev.2019.09.036 31580839

[47] Fischer B Russell C Sabioni P van den Brink W Le Foll B Hall W. Lower-risk cannabis use guidelines: a comprehensive update of evidence and recommendations. Am J Public Health. (2017) 107:e1–e12. 10.2105/AJPH.2017.303818 28644037 PMC5508136

[48] Vilches JR Taylor MB Filbey FM. A multiple correspondence analysis of patterns of CBD use in hemp and marijuana users. Front Psychiatry. (2021) 11:1583. 10.3389/fpsyt.2020.624012 33519562 PMC7840961

[49] Westman EC Behm FM Rose JE. Airway sensory replacement as a treatment for smoking cessation. Drug Dev Res. (1996) 38:257–262. 10.1002/(SICI)1098-2299(199607/08)38:3/4lt257::AID-DDR14&gt;3.0.CO;2-W7750331

[50] Westman EC Behm FM Rose JE. Dissociating the nicotine and airway sensory effects of smoking. Pharmacol Biochem Behav. (1996) 53:309–15. 10.1016/0091-3057(95)02027-6 8808137

[51] Naqvi NH Bechara A. The airway sensory impact of nicotine contributes to the conditioned reinforcing effects of individual puffs from cigarettes. Pharmacol Biochem Behav. (2005) 81:821–9. 10.1016/j.pbb.2005.06.005 15996724 PMC1434786

[52] Fischedick JT Hazekamp A Erkelens T Choi YH Verpoorte R. Metabolic fingerprinting of *Cannabis sativa* L, cannabinoids and terpenoids for chemotaxonomic and drug standardization purposes. Phytochemistry. (2010) 71:2058–73. 10.1016/j.phytochem.2010.10.001 21040939

[53] Booth JK Bohlmann J. Terpenes in *Cannabis sativa*- From plant genome to humans. Plant Sci. (2019) 284:67–72. 10.1016/j.plantsci.2019.03.022 31084880

[54] Casano S Grassi G Martini V Michelozzi M. Variations in terpene profiles of different strains of *Cannabis sativa* L. Acta Hortic. (2011) 925:115–21. 10.17660/ActaHortic.2011.925.15

[55] Tashkin DP Roth MD. Pulmonary effects of inhaled cannabis smoke. Am J Drug Alcohol Abuse. (2019) 45:596–609. 10.1080/00952990.2019.1627366 31298945

[56] Wei B Smith D O'Connor R Travers MJ Hyland A. Examining the association between body burdens of harmful chemicals and heaviness of marijuana smoking. Chem Res Toxicol. (2018) 31:643–5. 10.1021/acs.chemrestox.8b00160 30052028 PMC7083135

[57] Graves BM Johnson TJ Nishida RT Dias RP Savareear B Harynuk JJ. Comprehensive characterization of mainstream marijuana and tobacco smoke. Sci Rep. (2020) 10:7160. 10.1038/s41598-020-63120-6 32345986 PMC7188852

[58] Lanz C Mattsson J Soydaner U Brenneisen R. Medicinal cannabis: *in vitro* validation of vaporizers for the smoke-free inhalation of cannabis. PLoS ONE. (2016) 11:e0147286. 10.1371/journal.pone.0147286 26784441 PMC4718604

[59] Brezing CA Choi CJ Pavlicova M Brooks D Mahony AL Mariani JJ. Abstinence and reduced frequency of use are associated with improvements in quality of life among treatment-seekers with cannabis use disorder. Am J Addict. (2018) 27:101–7. 10.1111/ajad.12660 29457671 PMC5846101

[60] Liao J-Y Mooney LJ Zhu Y Valdez J Yoo C Hser Y-I. Relationships between marijuana use, severity of marijuana-related problems, and health-related quality of life. Psychiatry Res. (2019) 279:237–43. 10.1016/j.psychres.2019.03.010 30876731 PMC6713587

[61] Vergara D Bidwell LC Gaudino R Torres A Du G Ruthenburg TC. Compromised external validity: federally produced cannabis does not reflect legal markets. Sci Rep. (2017) 7:46528. 10.1038/srep46528 28422145 PMC5395929

[62] Zeyl V Sawyer K Wightman RS. What do you know about maryjane? A systematic review of the current data on the THC:CBD ratio. Subst Use Misuse. (2020) 55:1223–7. 10.1080/10826084.2020.1731547 32124675

[63] Jikomes N Zoorob M. The cannabinoid content of legal cannabis in washington state varies systematically across testing facilities and popular consumer products. Sci Rep. (2018) 8:4519. 10.1038/s41598-018-22755-2 29540728 PMC5852027

[64] Dei Cas M Casagni E Saccardo A Arnoldi S Young C Scotti S. The Italian panorama of cannabis light preparation: determination of cannabinoids by LC-UV. Foren Sci Int. (2020) 307:110113. 10.1016/j.forsciint.2019.110113 31927249

[65] Boehnke KF Gagnier JJ Matallana L Williams DA. Substituting cannabidiol for opioids and pain medications among individuals with fibromyalgia: a large online survey. J Pain. (2021) 22:1418–28. 10.1016/j.jpain.2021.04.011 33992787 PMC8578153

