# Supplementary material for: A “Good” Smoke? The Off-Label Use of Cannabidiol to Reduce Cannabis Use
Source: Front Psychiatry. 2022 Mar 17;13:829944. doi: 10.3389/fpsyt.2022.829944 (PMC8968154; doi:10.3389/fpsyt.2022.829944)
Supplement: Supplementary file 1 [file Table_1.DOCX]

**Supplementary Table 1: List of options to answer the question about primary reason for CBD use**

| To reduce the use of tobacco or other substances (illegal cannabis, alcohol, medicines, etc.) |
| --- |
| To save money on the use of illegal cannabis |
| To try the taste |
| To socialize |
| For my well-being |
| To have the effects of THC |
| To avoid the effects of THC |
| To be able to consume legal cannabis |
| To treat my illness or reduce related symptoms |
| Because I had difficulties obtaining illegal cannabis |
| Out of simple curiosity, with no particular expectation |
